# Contamination Evaluation and Source Analysis of Heavy Metals in Karst Soil Using UNMIX Model and Pb-Cd Isotopes

**DOI:** 10.3390/ijerph191912478

**Published:** 2022-09-30

**Authors:** Enjiang Yu, Hongyan Liu, Faustino Dinis, Qiuye Zhang, Peng Jing, Fang Liu, Xianhang Ju

**Affiliations:** 1 College of Resources and Environmental Engineering, Guizhou University, Guiyang 550025, China; 2 College of Agriculture, Guizhou University, Guiyang 550025, China; 3 Key Laboratory of Karst Georesources and Environment of Ministry of Education, Guizhou University, Guiyang 550025, China

**Keywords:** heavy metals, enrichment, source apportionment, isotopes, karst areas

## Abstract

Karst terrain is the typical area covered with a high background of heavy metals under geochemical anomaly. This research explored the accumulation of geochemical elements and soil sources in karst terrain from rock and soil exposed in carbonate areas. The comprehensive ecological risk and enrichment of heavy metals from parent rock weathered to soil was investigated in 11 formations in the carbonate and clastic areas of the Weining and Hezhang counties in northwest Guizhou. The single factor pollution index, geoaccumulation index, and the potential risk coefficient were used to assess the environmental risk. The results revealed that the heavy metals in an overall geologically high background level of soil in northwest Guizhou is at a slight risk level. However, except for Cd, the heavy metals did not exceed the standard pollution reference. Moreover, the UNMIX model and Cd and Pb isotopes were used to analyze the source of heavy metals, comprising of cadmium (Cd), arsenic (As), lead (Pb), chromium (Cr), copper (Cu), nickel (Ni), and zinc (Zn), and the geochemical elements of silicon (Si), aluminum (Al), iron (Fe), magnesium (Mg), and calcium (Ca). The study showed that most elements in the soil carbonate area exceed the national standard, and the heavy metals in the soil showed a strong enrichment, while the major elements Si and Mg display strong loss. Heavy metal concentrations in soil in the carbonate area were higher than in the clastic area. Geological sources and atmospheric deposition were the main contributors to heavy metal concentrations in both carbonate and clastic areas, and their concentrations differ according to soils developing in different formations.

## 1. Introduction

The global carbonate-exposed area accounts for approximately 12% of the land area [1,2]. In China, the southwestern province of Guizhou has a karst carbonate area of 15,104 km^2^, accounting for a third of the total land area [1], with typical geochemical high background characteristics. The northwestern region of Guizhou is recognized for the extreme developed carbonate strata (Guizhou Provincial Bureau of Geology and Mineral Resources, 2007). Wen et al. [3], reported that the karstic soil with a high geochemical background is significantly enriched with heavy metals; however, its bioavailability is generally low [3]. Meanwhile, an inheritance relationship in mineralogy and trace elements of geochemistry between the overlying soils and underlying carbonate rocks has been reported [4,5]. The concentration of metal elements in the overlying *terra rossa* (“red soil”) developed on karst landscapes is generally high, with most researchers concluding that the enrichment of heavy metals in soils was related to the parent rock’s content [3,6,7,8].

The enrichment of heavy metals in soils with higher carbonate development is much higher than in soils formed with parent rocks. This is mainly due to the special weathering formation process of carbonate rocks [3]. It has been suggested that the special weathering effect of carbonate rocks is one of the reasons for the significant enrichment of heavy metals during weathering carbonates [9]. The other reason is the secondary enrichment of elements after weathering of carbonate rocks [10].

The natural background of metals in terra rossa have been recognized to be primarily controlled by the lithology of parent rocks and are highly soil-type dependent [11]. Therefore, geographical or soil-type dependent distributions of metals can help to better understand their behaviors in terra rossa, owing to the complex and variable geological background and soil types across the study area. The accumulation of heavy metals in the soil is related to both the natural geological background and the influence of regional human activities, including agricultural activities such as pesticide spraying, fertilization, irrigation [12], industrial and mining activities [13,14], and urban residential activities [15]. Furthermore, in karst areas, the source of heavy metals in soil is more complex in areas with naturally high backgrounds and anthropogenic superposition [16].

Analysis of the sources of heavy metal pollution are essential for effectively and comprehensively controlling heavy metal soil pollution [17]. In recent years, various methods and models have been used to analyze the sources of heavy metals in soil, such as the geographic information system [11], multivariate statistical analysis [18], UNMIX models [19], positive matrix factorization (PMF) [16], and the chemical mass balance method (CMB). Among these methods and models, the UNMIX model is considered the simplest as its operation does not require complex tuning of the parameters or knowledge of the source component spectrum. The model is calculated based on the sample element concentration data, treating the concentration of the different elements as a linear combination of the components in the unknown source and assuming a positive contribution for each source. The UNMIX model has a species selection function which can remove anomalous data and generate graphs of the resolved source components and source contribution results using the system tool to generate plots. However, for these methods, a large number of samples are needed, and they are merely effective to identify the general pollution sources such as agricultural sources, industrial sources, and natural sources. It is limited in accurately identifying the exact origin of each pollution source and their contributions.

Stable isotope composition of heavy metals as a natural attribute and unique label, existing in different sources of substances, can effectively and accurately identify heavy metal pollution sources [6,16]. In order to better constrain and distinguish sources, lead (Pb) and cadmium (Cd) isotopes were used to quantify the source of heavy metals in this study. Pb has four naturally occurring isotopes (^204^Pb, ^206^Pb, ^207^Pb, and ^208^Pb). The different Pb sources may have characteristic isotopic compositions, so that variations of Pb isotope ratios in the environmental media can be used to determine the origins of Pb and contributions of natural or anthropogenic sources, such as sediments [20], topsoils [21], plants [22], and so on. Cd has eight stable isotopes (^106^Cd, ^108^Cd, ^110^Cd, ^111^Cd, ^112^Cd, ^113^Cd, ^114^Cd, and ^116^Cd) and the relative molar proportions vary between 0.89% for ^108^Cd and 28.17% for ^114^Cd [23]. Different sources of samples showed differences in Cd isotopic values. Therefore, such fractionated Cd can be used to trace the sources of Cd in the environment [6].

At present, several investigations are focusing on relatively large regional ranges of the geochemical features of the explored elements [24,25,26]. Regional soils with single lithology are rarely investigated due to the complexity of lithology and/or the significant contribution of anthropogenic pollution in the large regions being explored. In this study, a systematic rock and soil sampling survey and elemental analysis were conducted to explore the accumulation of geochemical elements in the karst area in the northwest region of Guizhou. The geochemical elements and the accumulation rules of rock and soil were expressed using mathematical models and statistical methods. Furthermore, the sources of the soil’s geochemical elements were analyzed using a correlation analysis, a Pb and isotope binary model, and an UNMIX model. This research provides scientific data and theoretical support for the prevention and control of excessive heavy metals in soil in the karst region.

## 2. Materials and Methods

### 2.1. Study Area

**Figure 1:**
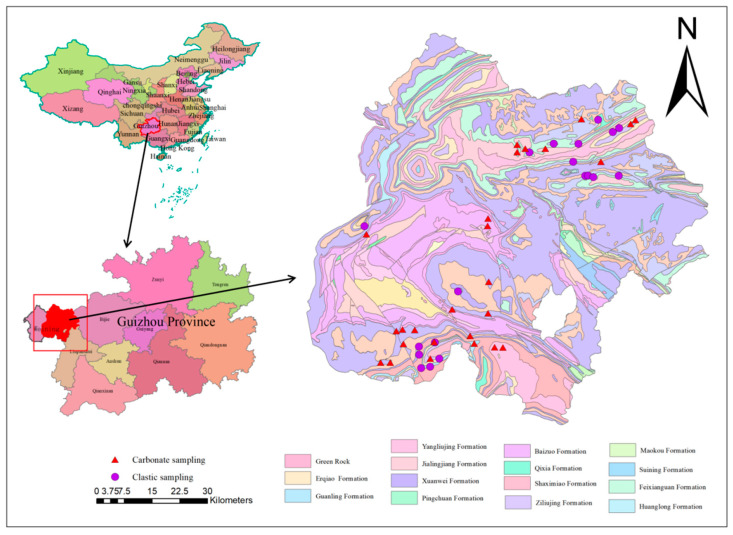
Location of sampling sites in Guizhou Province, China.

The study area was determined via GPS (Trimble GEO 7X, Shanghai Navearth, Shanghai, China). The study area is shown in Figure 1. The study area is located northwest of Guizhou Province, comprising of Weining County and Hezhang County (103°36′–104°45′ E, 26°36′–27°26′ N) and (104°10′–105°01′ E, 26°46′–27°28′ N), respectively. The average altitudes of Weining and Hezhang are 2200 and 1996 m, with areas of 6295 and 3250 square kilometers, respectively. Weining County has a high altitude, plateau platform, and a low latitude. It has 4 clear seasons, a warm spring, and is less cold in winter due to its climate characteristics. It is geologically located in the secondary tectonic units with a complete formation. It has a complex structure and a wide distribution of volcanic rocks. In the study area, the parent rocks are carbonate rocks and clastic rocks. The clastic sub-area is comprised of Erqiao, Feixiangguan, Longtan, and Xuanwei formations. The carbonate sub-area included Suining, Ziliujing, Guuanling, Jialingjiang, Maokou, Maping, and Huanglong formations (Table 1 and Figure 1.)

### 2.2. Sample Collection

The exposed rock and soil were collected and based on all stratum in the study area.

#### 2.2.1. Rock Samples

Rock samples were collected along roads or canals within the outcrops of freshly exposed rock. The rock sampling included various kinds of formations in this study, which totaled 69. Subsequently, the rock samples were broken in a jaw crusher, further crushed in a roll crusher, and pulverized in a ball mill into 200 mesh (<0.074 mm) for chemical analysis.

#### 2.2.2. Soil Samples

For the study, soil samples were collected in September 2019 at the designated sites. The sample is the developing soil or the wasteland soil around the rock sampling site. 60 soil samples were collected with a probe instrument. Each sample was collected from the top 20 cm of soil at the designated sites. The samples were then air-dried, ground, and passed through a 20-mesh (<0.84 mm) nylon sieve. Four soil sub-samples were combined to make a composite soil sample.

### 2.3. Sample Analysis

#### 2.3.1. Soil Samples

Each soil sample was analyzed to determine the total concentration and pH value of 12 components (Cd, As, Pb, Cr, Cu, Ni, Zn, SiO_2_, Al_2_O_3_, TFe_2_O_3_ (Total Fe as TFe_2_O_3_), MgO, and CaO). Chemical analyses were conducted at the testing center of the College of Resources and Environmental Engineering, Guizhou University, subsequent to pre-treatment using the specifications of the multi-purpose regional geochemical survey [27]. The measurement methods to determine the total content of elements depend on elemental types. For instance, SiO_2_, Al_2_O_3_, TFe_2_O_3_, MgO, CaO, and K_2_O were measured with X-ray fluorescence spectroscopy (XRF, PANalytical Axios; RIGAKU ZSX Priums, Tokyo, Japan), Cd, As, Pb, Cr, Cu, Ni, and Zn measured with inductively coupled plasma mass spectrometry (ICP-MS). Additionally, the pH value was measured using the anion-selective electrode method.

Fresh chips of the sample were hand picked and a standard volume of chips (approximately 28 g) was ground in a swing mill with tungsten carbide surfaces for 2 min. Three and a half grams (3.5 g) of the sample powder was weighed into a plastic mixing jar with 7.0 g of spec pure dilithium tetraborate (Li_2_B_4_O_7_) and, assisted by an enclosed plastic ball, mixed for 10 minutes. The mixed powders were emptied into graphite crucibles with internal measurements of 34. 9 mm diameter by 31.8 mm deep. Twenty-four (24) filled crucibles were placed on a silica tray and loaded into a muffle furnace only large enough to contain the tray. Fusion took 5 min from the time the preheated furnace returned to its normal 1000 °C after loading. The silica plate and graphite crucibles were then removed from the oven and allowed to cool. Each bead was reground in the swingmill for 35 s, and the glass powder was then replaced in the graphite crucibles and refused for 5 min. SiO_2_, Al_2_O_3_, TFe_2_O_3_, MgO, CaO, and K_2_O were measured with X-ray fluorescence spectroscopy (XRF, PANalytical Axios; RIGAKU ZSX Priums).

Soil samples of 0.1 g were weighed and placed into the polytetrafluoroethylene inner tanks of the digestion kettle through a 100-mesh sieve. Three mL HNO_3_ (guarantee reagent) and 3 mL HF (guarantee reagent) were added, and the sample was left for 8 h. Following this, 2 mL of HClO_4_ (guarantee reagent) was added and placed in the metal outer tank of the digestion kettle to dissolve in the oven at 180 °C for 12 h. When the digestion was complete, the inner polytetrafluoroethylene tanks on the electric hotplate were heated to completely dry the acid and remove any HF residue. One mL of HNO_3_ (guarantee reagent) was added to dissolve and the volume of 3% diluted HNO_3_ (guarantee reagent) was fixed to 50 mL before the heavy metal (Cd, As, Pb, Cr, Cu, Ni, and Zn) concentrations were determined by inductively coupled plasma mass spectrometer (ICP-MS; Thermo Fisher Scientific X2, Waltham, MA, USA).

#### 2.3.2. Rock Samples

Rock samples were analyzed to ascertain the total concentration of 14 components (Cd, As, Pb, Cr, Cu, Ni, Zn, SiO_2_, Al_2_O_3_, TFe_2_O_3_, MgO, and CaO). SiO_2_, Al_2_O_3_, TFe_2_O_3_, K_2_O, Cr, and Zn were measured with XRF, while As and Hg were measured using AFS, and Cd, Ni, and Pb by ICP–MS; CaO, MgO, Cu, and Mn were measured by ICP–OES.

#### 2.3.3. Quality Assurance and Quality Control (QA/QC)

Standard reference materials (GSS–5 for soil samples and GSR1–6 for rock samples) were used to validate the accuracy and precision of the analytic methods. The analytical quality control showed a good precision, with the relative standard deviation being <8.50%.

### 2.4. Assessment of Soil Contamination

#### 2.4.1. Enrichment and Loss Assessment of Elements in Soils

The enrichment coefficient (*EF*) is often used to indicate the degree of enrichment of an element relative to its natural source [28]. For instance, Bergamaschi et al. and Ding and Ji have used this to evaluate the degree of enrichment (contamination) of metal elements [29,30]. The *EF* value of component *i* in soil can be calculated using:

*EF_i_* = (*C_i_*/*C_n_*)_sample_/(*C_i_*/*C_n_*)_baseline_ (1)

where *C_i_* is the concentration of component *i*, and *C_n_* is the concentration of the standardizing element *n*. Elements with stable geochemical properties, such as Al, Ti, Sc, Y, and Zr, are commonly used as standardized reference elements in epigenetic geochemical processes [29,30]. In this study, Al was adopted as the reference component *n*. Previous studies revealed that Al is inert in the epigenetic geochemical process, thus suggesting that Al is a non-mobile element [10]. The soil samples were taken as the sample, and the rock samples were taken as the baseline in this study. Thus, the formula for calculating the *EF* value of component *i* are given below as:

*EF_i_* = (*C_i_*/*C*_Al_)_soil_/(*C_i_*/*C*_Al_)_rock_ (2)

*Q* index is applied to determine the degree of accumulation or loss of elements in the weathering process, and is calculated as follows [31]:

*Q_i_* = *C_i_*_-soil_ /*C_i_*_-rock_ (3)

where *C_i-_*_soil_ and *C_i-_*_rock_ are the concentration of component *i* in soil and rock, respectively.

#### 2.4.2. Single-Factor Pollution Index

The PI calculation method expressed by the formula:

(4) $$P_{i}=C_{i}/S_{i}$$

where *C_i_* represents the concentration of heavy metal *i* in tailing wastes and *S_i_* denotes the background concentration of heavy metal *i*. The background values [32] were selected following the techniques and considered as reference values for evaluation of soil pollution (see Table 1).

#### 2.4.3. Geoaccumulation Index

Geoaccumulation indexes (*Igeo*) for metal content were determined using Muller’s (1979) equation:

(5) $$\mathrm{Igeo}=\log_{2}(\frac{C_{i}}{1.5\times B_{i}})$$

where *C_i_* is the measured concentration of the *i* heavy metal examined in the soil, *B_i_* is the background value of the elements in the soil [32], and the factor 1.5 was used to correct possible variations in the background values of the specific metal in the environment and anthropogenic influences (see Table 1).

#### 2.4.4. Potential Ecological Risk Index

The monomial potential ecological risk factor (*E_i_*) and the comprehensive ecological risk index (*RI*) were applied for the heavy metal risk assessment [33]. *E_i_* and *RI* are given by the expression:

(6) $$E_{i}=p_{i}\times T_{i}$$

(7) $$RI=\sum E_{i}$$

where *P_i_* is the single contamination index of metal *i* calculated by Equation (1), and *T_i_* represents the metal toxicity response coefficient of metal *i*. The toxicity response factors for Cd, As, Pb, Cr, Cu, Ni, and Zn are 30, 5, 5, 2, 5, 5, and 1 [33], respectively (see Table 2).

#### 2.4.5. Pb and Cd Isotopic Composition Analysis

##### Pb Isotope Analysis

The Pb IRs were measured using a sector magnetic field plasma mass spectrometer (HR-ICP-SFMS) at the ALS Chemex Co., Ltd., (Guangzhou, China). An amount of aliquot powder was dissolved in a mixture of concentrated HF and concentrated HClO_4_ for 72 h. The Pb in the digested sample was separated and purified using conventional anion exchange techniques (200–400 mesh AG1 × 8 resin) and diluted HBr. The ^207^Pb-^204^Pb double spike method was employed to correct the mass fractionation effects for the Pb isotopic analysis. When 1 µg of Pb was analyzed, the within-run analytical precision of the ^208^Pb/^206^Pb IR was below 0.005%. Repeat analysis of reference material NBS981 gave a ^208^Pb/^206^Pb IR of 2.1652465 ± 0.000069, a ^207^Pb/^206^Pb IR of 0.9145100 ± 0.000056, and a ^204^Pb/^206^Pb IR of 0.0591995 ± 0.000013.

##### Cd Isotope Analysis

The Cd IRs were measured using a multi-collector inductively coupled plasma-mass spectrometer (MC-ICP-MS) at the ALS Chemex (Guangzhou) Co., Ltd., China. Firstly, approximate sample powders containing 380 ng of Cd were weighted into Teflon bombs and digested with a mixture of concentrated HF and HNO_3_ with a volume ratio of 1.5:1. After heating in the oven at 190 °C for 3 days to completely break down the silicates, refractory minerals, and organic materials, the samples were transferred into Savillex screw-top beakers. The samples in beakers were then evaporated to dryness and a 3:1 (*v*/*v*) mixture of concentrated HF and HNO_3_ was added into the samples, then placed on the hot plate, and heated at 140 °C for 2 days. The samples in the beakers were evaporated to dryness again, and aqua regia was added. Following this, the samples were heated at 100 °C until fully dissolved. Subsequently, the samples were evaporated to dryness, followed by treatment with concentrated HCl. Volumes of the double spike (^111^Cd–^113^Cd) were added to the samples to avoid Cd isotope fractionation caused by the chemical procedure. Finally, the samples were evaporated to dryness and dissolved in 2 mL 6 mol/L HCl for ion-exchange chromatography.

Chemical purification of Cd was performed using a one-step chromatography with 2 mL anion exchange resin (AG1-X8 resin, 100–200 mesh, Bio-Rad, Hercules, CA, USA). Matrix elements Na, Mg, Ca, Al, Ti, and Zr were eluted with 6 mol/L HCl (4 mL). Fe, Ga, Ag, Pd, Mo, and In were then eluted with 0.3 mol/L HCl (25 mL). Following this, Zn and Sn were eluted with 0.5 mol/L HNO_3_ + 0.1 mol/L HBr (30 mL), and Cd was collected with 2 mol/L HNO_3_ (10 mL).

The whole procedural blank for Cd was <1 ng, which is negligible compared with the amount of Cd (>400 ng) loaded on the column. Cd isotope compositions were measured using the double spike method on an MC-ICP-MS. The typical sensitivity of ^114^Cd was ~120V/ppm using an Aridus II desolvator (Teledyne CETAC Technologies, Omaha, NE, USA) in low-resolution mode. The cup configuration was set as ^110^Cd, ^111^Cd, ^112^Cd, ^113^Cd, ^114^Cd, and ^116^Cd, and collected on L3, L2, L1, C, H1, and H2 Faraday cups, respectively. ^105^Pd, ^115^In, ^117^Sn, and ^120^Sn were also measured to precisely correct the interferences of ^110^Pd, ^113^In, and ^114^Sn. Cd isotope data are reported in a δ notation in per mil against the international reference material NIST CRM 3108 [34].

(8) δC114/110d=((C114dC110d)sample(C114dC110d)standard-1)×1000(‰)

The data quality of the Cd isotope analysis was rigorously monitored using a number of in-house standards, international reference materials, and duplicated samples. The long-term external precision of δ^114^/^110^Cd was monitored by the analysis of pure Cd solution BAM I012 Cd (δ^114/110^Cd = −1.325 ± 0.043‰, *n* = 74, 2SD), Münster Cd (δ^114/110^Cd = 4.455 ± 0.047‰, *n* = 71, 2SD), and AAS Cd (δ^114/110^Cd = −0.691 ± 0.041‰, *n* = 57, 2SD) over 7 months, and was found to be better than ±0.050‰. The δ^114/110^Cd of international soil standards NIST SRM 2711 (0.643‰) are in good agreement with the literature values (0.630–0.803‰) [35]. The reproducibility of δ^114/110^Cd for soil standards are better than 0.025‰ (2SD), and duplicate samples are better than 0.08‰ (2SD). These results showed that the Cd isotope data are reliable.

### 2.5. Statistical Analysis

#### 2.5.1. UNMIX Model

The UNMIX model was performed using the US EPA (Washington, DC, USA) UNMIX6.0 software. The UNMIX model has a strict data screening process based on an eigenvalue analysis. The missing data or data below minimum detection limits are eliminated. Results from the UNMIX model are constrained to nonnegative values since geometrical concepts of self-modeling curve resolution are used. Moreover, using the singular value decomposition (SVD) method, the model estimates the source number by reducing the dimensionality of data space *m* to *p* [36,37].

The calculation formula is as follows:

(9) $$c_{ij}=\sum_{k=1}^{m}F_{jk}S_{jk}+E$$

where *C_ij_* is the content of elements in the *j* th species of the i th regional sample (soil); *F_jk_* is the *j* th sample element content in source *k* (*k* = 1, … m), representing the composition of the source; *S_ik_* represents the total amount of source *k* in the *i*th sample, that is, the contribution rate of the source; and *E* is the uncertainty of the analysis process or the standard deviation of each source composition. In this study, in order to eliminate the impact of large differences between different trace heavy metals in the soil, the deviation normalization method was used to process all the data within 0–1 before inputting the data into the UNMIX model. The calculation formula is as follows:

(10) $$X_{k}=\frac{X_{i}-MinX_{i}}{MaxX_{i}-MinX_{i}}$$

A total of 12 heavy metal elements including Cd, As, Pb, Cr, Cu, Ni, Zn, SiO_2_, Al_2_O_3_, TFe_2_O_3_, MgO, and CaO in 60 soil sampling points were standardized by the method of deviation standardization. The standardized data were dimensionless, and the value range of variable observation values would be between 0 and 1. The standardized data are substituted into UNMIX 6.0 software for analysis.

#### 2.5.2. Pb and Cd Isotopic “Binary Model”

The source contribution is calculated using the isotope “binary model” formula, and the equations used in this study are as follows:

(11) $$f_{1}+f_{2}=1$$

(12) f1×P206b/P207bi1+f2×P206b/P207bi2=P206b/P207bi3or f1×P208b/P206bi1+f2×P208b/P206bi2=P208b/P206bi3or f1×C114d/C110di1+f2×C114d/C110di2=C114d/C110di3

where f is the contribution proportion of the fraction, P206b/P207b, P208b/P206b, and C114d/C110d are the isotope ratio, $i_{1}$ is the geological source, $i_{2}$ is the atmospheric deposition source, and $i_{3}$ is the topsoil.

The experimental area was obtained from a satellite map, measured onsite via GPS (Trimble GEO 7X, Shanghai Navearth, Shanghai). Descriptive statistical analyses and plotting of figures were carried out using Origin 9.0 (Origin Lab Corporation, Northampton, MA, USA) and Excel 2013 (Microsoft Corp., Waltham, MA, USA). Summary statistics were used to calculate the average values, standard deviations, and analysis of variance using SPSS version 22.0. Principal component analyses were conducted to compare different elements of samples from the same site. A geochemical element source analysis is one of the source analysis models recommended by the US Environmental Protection Agency (EPA).

## 3. Results and Discussion

### 3.1. Elemental Geochemical Characteristics in Rocks and Soils

#### 3.1.1. Rocks

The heavy metal concentration of rock in different formations is shown in Table S1. The highest concentration of Cd in the Feixianguan formation in the clastic sub-area was 0.54 mg/kg. This value was 1.5–4.5 times greater than the rock abundance value in northwest Guizhou [38]. On the contrary, the lowest Cd content of mapping formation in the carbonate sub-area was 0.12 mg/kg, which was 1.0–3.3 times greater than of the rock abundance value in northwest Guizhou. Overall, the Cd concentration of rock was higher in the clastic sub-area than in the carbonate sub-area. A summary of major and trace element concentrations of rocks is shown in Table 3.

Generally, in the carbonate area, the mean concentrations of MgO_2_ and CaO_3_ in the rocks in northwest Guizhou were considerably lower than other abundance values of carbonate rocks in China [38], while the remaining nine element concentrations presented an opposite trend. The Cd concentration of the studied carbonate rocks was (0.42 mg/kg), which is close to the value obtained for limestone in Konya, Turkey (0.400 mg/kg, [39]. It is worth pointing out that the global average limestone concentration is 0.028 mg/kg [40]. In comparison, the rock heavy metal concentration in the clastic rock area is higher than that of the carbonate area.

#### 3.1.2. Soils

Statistics of heavy metals in soils of different strata are shown in Table 4. The highest concentration of Cd in the Longtan formation in the clastic sub-area was 7.66 mg/kg, and the lowest Cd content of the Ziliujing formation in the carbonate sub-area was 0.49 mg/kg. Statistical results of total concentrations of 14 components in soils are shown in Table 5. In the carbonate area, the soil pH median was 6.53; the average pH of 6.43 suggested that the topsoil in the study area was weakly acidic. The average concentrations of Cd, As, Pb, Cr, Cu, Ni, Zn, TFe_2_O_3_, MgO, Al_2_O_3_, and CaO in the soils were considerably higher than other soils in China [32]; however, the SiO_2_ concentrations presented an opposite trend. In the clastic sub-area, the soil pH median was 6.25; the average pH of 6.43 suggested that the topsoil in the study area was weakly acidic. The average concentrations of Cd, Pb, Cr, Cu, Ni, Zn, TFe_2_O_3_, MgO, and Al_2_O_3_ in the soils were considerably higher than other soils in China. Similarly, it was found that SiO_2_ and CaO concentrations were lower than other soils in China. This finding reflects the unique characteristic of the enrichment of heavy metals in karstic soil with a high geochemical background under the same geological condition [10]. The concentration distribution of heavy metals in soil in the study area showed that the heavy metal concentration in the carbonate area was significantly higher than that in the clastic area. The Cd mean concentration in the carbonate substrate topsoil was 1.90 mg/kg, which is significantly higher than the Cd concentration in clastic sub-area (1.61 mg/kg).

The coefficient of variation (CV) reflects the degree of discretization between the data and the size of spatial variability in the samples. It is generally believed that a CV of less than 10% represents a weak variation, while variations at 10–100% signify moderate variation, and greater than 100% shows strong variation [17]. In the carbonate area (Table 5), the CV of all elements except for Cd were moderate variations (10–100%). The CV for Cd was 125.74%, which is a strong variation, indicating that the source of soil Cd was extensive and may be greatly affected by human activities. The CV of pH was lowest (14.00%). The findings further revealed that As, Ni_,_ Zn, Al_2_O_3_, and Fe_2_O_3_ demonstrated strong variation in the clastic sub-area. The CV of Ni was highest (262.53%). In general, all elements were of moderate to high variability, indicating that the variability of elements in soils throughout the study area was high, and the degree of spatial dispersion was large, which was likely to be affected by human activities [17]. Furthermore, compared with the carbonate area, the CV of the soil in the clastic sub-area is significantly higher and more affected by exogenous heavy metals to a certain extent, particularly the Ni content.

### 3.2. The Enrichment or Loss of Elements in Formation Process of the Soil-Forming Process

#### 3.2.1. Relative to the Background Value

To compare elemental compositions and the enrichment or loss of each element during the weathering process in the carbonate, the enrichment coefficient (*EF*) was used in this study. This was obtained by dividing the concentration of each element in the soil by the background value in northwest Guizhou [32]. The normalized elemental compositions of soils in the carbonate are shown in Table 6.

For major elements in soil from the carbonate sub-area, the *EF* of Si was less than 1.0, which indicates that the elements were lost during weathering. Fe and Al had *EF* values between 0.95 and 1.05 and did not show enrichment during weathering. For other elements, the *EF* value is greater than 1.05 and shows a strong enrichment during weathering [10,29]. For trace elements, Cd and Zn revealed strong enrichment in soils during weathering with an *EF* of more than 1.4, while As was lost, with an *EF* value of 0.91. These values are similar with the enrichment values reported by Yang et al. (2021). The normalized value of Cd in the elements is 4.22, showing a strong enrichment state, which is consistent with Luo et al. (2018) [41]. Elemental accumulations and losses were significantly different, and the *EF* values varied between 0.78 and 3.23. Therefore, it can be inferred that the high cadmium concentrations in carbonate soil are associated with the high prevalence of Cd inherent in carbonate rocks, and that residual enrichment during carbonate weathering increases concentration effects.

Many of the elements showed similar trends to carbonate rocks in the clastic area with the exception of Ca, which showed a strong loss of (*EF* = 0.92). Furthermore, except for As, the trace and heavy metal enrichment in soils during weathering revealed EF values of more than 1.4. However, the elements Pb, Cr, Cu, Ni, and Zn relatively enriched in the soils from the carbonate substrates during weathering, and strong enrichments were observed in soils from the clastic sub-area.

#### 3.2.2. Relative to the Bedrocks

In order to show the elemental difference between carbonate rocks in northwest Guizhou and the average carbonate rocks in China herein, *Q* was used which was obtained by dividing the concentration of each element in carbonate rock by the average carbonate abundance in China [38]. The comparison data shown in Table 6 revealed that metal elements such as Pb, Cu, Ni, Al, and Ca had a Q value of less than 1.0 in carbonate rock from Guizhou. These concentrations are lower than the average concentrations from carbonate rocks in China. The *Q* values of Cd, Cr, Zn, Fe, and Mg were all higher than 1.4, showing the characteristics of high carbonate content in northwest Guizhou compared with average concentrations of the metals in carbonate rocks in China. These values are suggestive of the geochemical abnormality of heavy metals in the soil in northwest Guizhou. For Si and Al elements, *Q* values were approximately 1 during rock weathering, exhibiting stable geochemistry, and the elements are inert in the epigenetic geochemical process [29].

The *Q* values of Al and Fe were 1.60 and 1.53, indicating that the accumulation of Al and Fe was significant and the degree of aluminization and ferritization were extremely high in the carbonate sub-area. Compared with the *Q* values reported by Yang et al. (2021), the range of *Q* values in the carbonate region varies widely between 0.01 and 94.72, and the degree of aluminization and ferritization was extremely high (the *Q* values of Al and Fe were 82.61 and 80.14). In clastic soil, Si elements showed a loss. Multiple elements (e.g., As and Pb) exhibit loss in the clastic rock area compared with the carbonate area. Furthermore, elemental accumulations and losses in the clastic area were wide (0.20–11.38) compared with the carbonate area. This finding indicates that elemental accumulation or loss in the clastic area showed relative activity.

### 3.3. Contamination Evaluation of Heavy Metal Pollution in Soil

#### 3.3.1. Single Factor Index and Geoaccumulation Index

The single factor index method assumes the soil environmental background value [32] as the evaluation standard to assess the heavy metal pollution in the developed soil in karst areas. The single factor pollution index of each heavy metal and the geoaccumulation index of the carbonate sub-area and clastic sub-area in the study area were obtained. Table 7 presents the proportion of heavy metal pollution in the soil of the seven heavy metals. The pollution grade of the soil heavy metal Cd was the highest, with the element reaching a pollution level of 65.63% in the carbonate sub-area. The seven elements exceeded the standard rates in order of Cd > Pb > As > Cr = Cu = Zn > Ni, with Pb and As reaching 21.88% and 18.75%, respectively. Apart from Cd which showed serious accumulation and enrichment in the soil, the remaining six heavy metals showed relatively small accumulation and enrichment.

The Cd content reached a slight pollution level of 57.14% in the clastic sub-area. The order of pollution for the seven elements in the clastic sub-area was Cd > Cu > Zn > Ni > Cu > Cr > Pb > As, with Cu, Zn, and Ni beyond level four, accounting for 42.86%, 35.71%, and 32.14%, respectively. This indicates that the accumulation and enrichment of Cd, Cu, Zn, and Ni in soil is serious. Compared with the single factor pollution index in the carbonate rock area, the accumulation degree of Cd is lower. The geoaccumulation index of seven heavy metal elements in the study area (Table 6) is relatively low. Except for the clastic sub-area Cu, all the other heavy metals are not exceeding the standard. The local accumulation index of Cu in the clastic area was 28.57%.

Based on the single factor pollution index and the geoaccumulation index, the Cd at all points in the study area exceeded the standard pollution reference, which may be associated with the secondary accumulation of heavy metals during rock weathering [10,29]. This was related to the large toxicity coefficient of Cd [42]. The potential ecological risk coefficient indicates that the overall geological high background development soil in northwest Guizhou is at a slight risk level. This study highlights that a comprehensive and reliable assessment of the status of soil heavy metal contamination in rock weathering development soils is imperative for environmental monitoring, remediation, and decision making.

#### 3.3.2. Potential Ecological Risk Index

**Figure 2:**
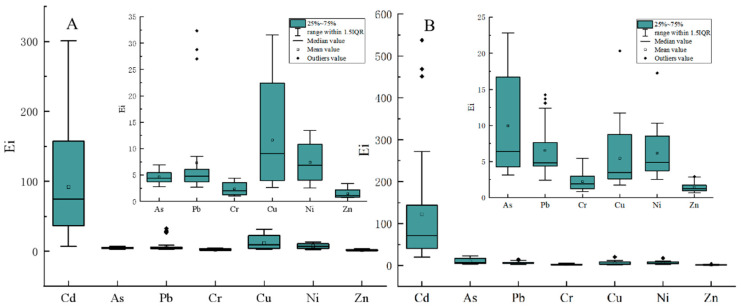
Evaluation of the individual element potential ecological risks of heavy metals. (**A**) carbonate soils and (**B**) clastic soils.

**Figure 3:**
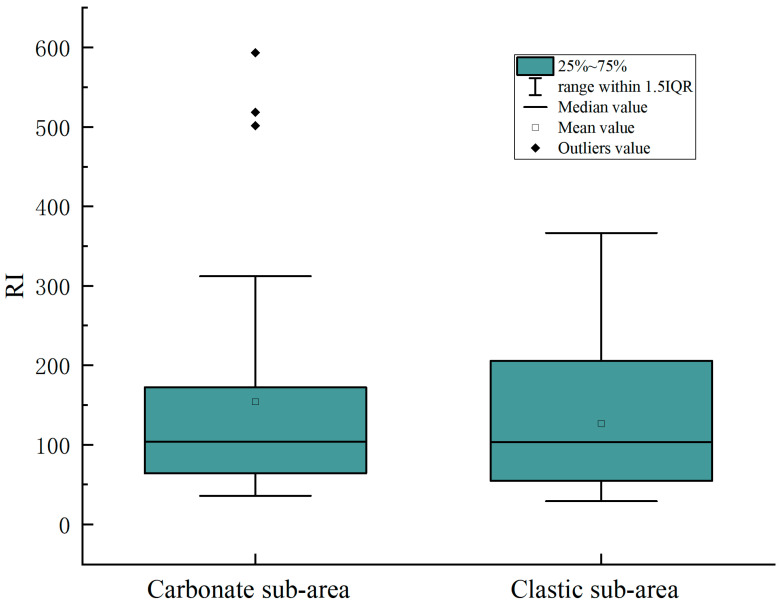
Evaluation of the potential ecological risks of heavy metals.

The *Ei* values of the potential ecological risk coefficient of individual heavy metals are shown in Figure 2. The average potential ecological risk coefficient of the seven heavy metals were Cd > Cu > Ni > Pb > As > Cr > Zn in the carbonate sub-area. The total potential ecological risk coefficient of Cd in the soil was between medium risk and high risk, representing 85.00%. This may be related to the large toxicity coefficient of Cd [42]. The potential ecological risk coefficient of the remaining six heavy metals was less than 40, which is a slight risk to the soil ecological environment. The mean potential ecological risk coefficients for the seven heavy metals in the clastic sub-area were Cd > As > Pb > Cu > Ni > Cr > Zn. The total potential ecological risk coefficient of Cd in soil ranked between medium risk and higher, which accounts for 67.86%. The potential ecological risk coefficient of all samples of the remaining six heavy metals was less than 40, which is a slight risk to the soil ecological environment. Moreover, as shown in Figure 3, the environmental background value of soil in northwest Guizhou was taken as the evaluation standard, the average RI value of soil in the carbonate area was 153.94, and the rate exceeding the slight risk degree was 25.00%. The average RI value of the soil in the clastic sub-area was 126.78, and the ratio over the slight risk level was 28.57%, indicating that the overall performance of heavy metals in the developing soil in the study area was at a slight risk level.

The environmental background value of soil in northwest Guizhou was taken as the evaluation standard, the average RI value of soil in the carbonate area was 153.94, and the rate exceeding the slight risk degree was 25.00%. The average RI value of the soil in the clastic sub-area was 126.78, and the ratio over the slight risk level was 28.57%, indicating that the overall performance of heavy metals in the developing soil in the study area was at a slight risk level.

### 3.4. Source Analysis of Heavy Metals

#### 3.4.1. Correlation Analysis

The major components of soils such as Al, Fe, clay minerals, and organic matter play an important role in the distribution of heavy metals [10]. The relatively stable Al, Fe, and Mn oxides are widely distributed in soils in tropical and subtropical regions [43], where the degree of weathering is high and the clay content in soil is rich. In order to study the factors affecting the accumulation of metal elements in soils of the study area, the Pearson correlation coefficient between soil pH, Al_2_O_3_, TFe_2_O_3_, MgO, CaO, and elements (As, Cd, Cr, Cu, Hg, Ni, Pb, and Zn) in soils from the carbonate were calculated [17,44]. The results are shown in Table 8.

The pH value significantly affects the geochemical behavior of the elements in the soil [45,46]. In Table 8, the pH values were negatively correlated with As, and over a certain range showed a significantly positive correlation with Ca. The pH values showed a negative correlation with metal elements and a positive correlation with alkaline metals. The sorption of the metal Fe minerals, such as hematite and goethite, and Al minerals, such as gibbsite, increases gradually with the increase in pH [10,45]. Moreover, in the case of high pH, cationic metals generate multinuclear polymers or form precipitate on gibbsite [47,48]. Consequently, the effects of Al_2_O_3_ and TFe_2_O_3_ on the accumulation of heavy metals in karstic soils were particularly significant. For instance, Zn showed a significant positive correlation with Cd, Pb, Cr, and Ni (*p* < 0.01), and the correlation coefficient was greater than 0.5. Furthermore, Cd displayed significant positive correlations with As and Zn (*p* < 0.01) and Pb and Ni (*p* < 0.05).

Elements with significant correlations may have a strong homology or similar deposition mechanisms, indicating that the seven heavy metal elements may have similar sources and are simultaneously affected by multiple sources [17]. Four major elements, Ca, Cr, Zn, and Mg, presented a significant positive correlation (*p* < 0.01), and the correlation coefficient was greater than 0.4. Fe along with Cr, Cu, and Ni showed a significant positive correlation (*p* < 0.01), and the correlation coefficient was greater than 0.5. Considering the elemental content in soils, the stability of Ca and Fe indicated that geological sources influenced the increase in each element.

#### 3.4.2. Source Apportionment by UNMIX Model

In order to understand the source contributions of 11 elements, 60 samples were analyzed using UNMIX 6.0 software. The results revealed the Min Rsp = 0.83 was greater than the system required minimum (Min Rsp > 0.8), signifying 83% of the species variance. Additionally, Min (Sig/Noise) = 3.44 is greater than the system required minimum (Min (Sig/Noise) > 2), and the analytical results from these two sources are found to be credible.

**Figure 4:**
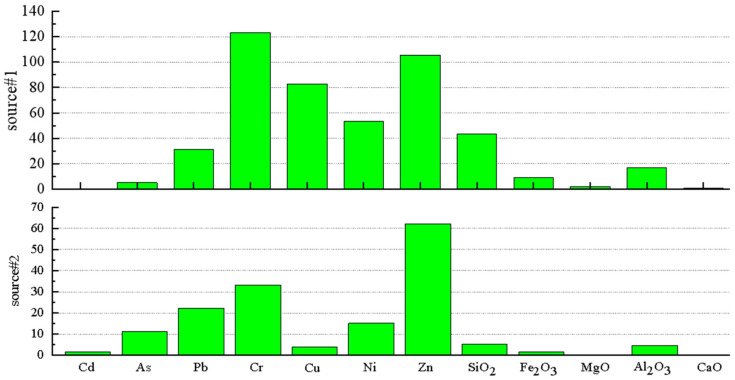
Source component profiles of geochemical elements in soil with UNMIX.

**Figure 5:**
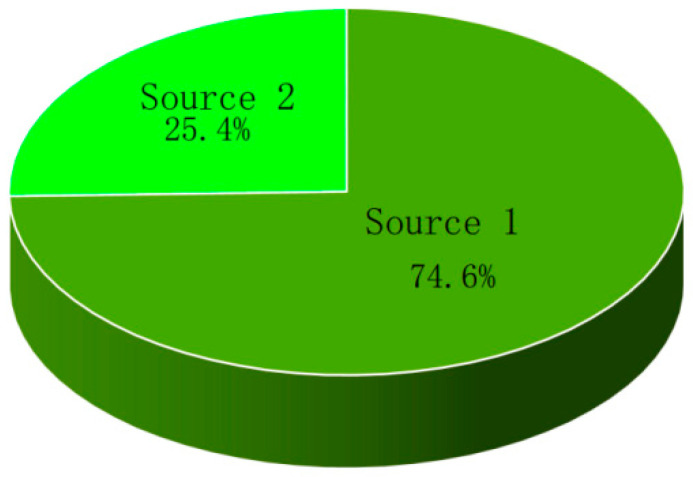
Distribution of source contributions in soil with UNMIX.

**Figure 6:**
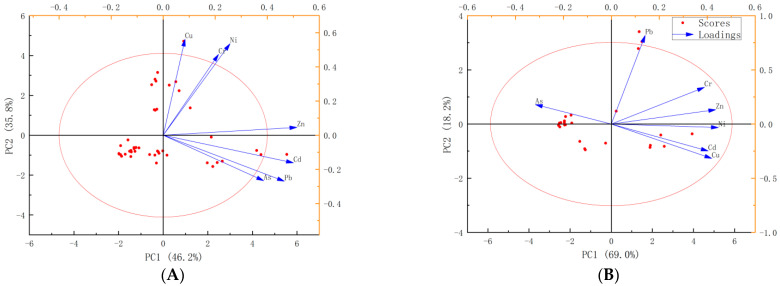
Principal component analysis of heavy metals in soil. (**A**) carbonate soils and (**B**) clastic soils.

Source component profiles of geochemical elements in soil are shown in Figure 4. The contribution of source one, Cr, Zn, Cu, and Ni to the soil were predominant. The heavy metal accumulation coefficient shows accumulation from multiple sources. The distribution of source contributions of geochemical elements in soil is shown in Figure 5. The contribution proportion of source one was 74.60%. In this region, Cr, Ni, and Zn are geological sources and electroplating processes. [49,50,51,52]. Analysis of the principal component (Figure 6) revealed that the amounts of Cr and Ni in the study area were large, and comparison with the background value presented no evidence of significant accumulation of concentration content, thus suggesting that Cr and Ni mainly came from the secondary enrichment after rock weathering [11,16]. In source one, the major elements Al and Si were dominant in the source contribution of the soil. Al was identified as a conservative element; it is the third most abundant crustal element and is believed to originate largely from the crust [44]. Northwest Guizhou is located in the geological high background area of heavy metals Zn and Cr, and geological anomalies are characteristic of the Karst region [53,54]. There was no industrial activity around the sampling site, which means that the comprehensive analysis of source one revealed that the geological sources and parent rock weathering were the contributing sources of soil geochemical elements. The elements Zn and Cr were dominant in the soil contribution of source two. The contribution proportion of source two was 25.40. From 1980 to 2004, the site was used for large-scale artisanal zinc smelting. The exhaust gas caused heavy metal pollution through atmospheric deposition during zinc smelting [41,55], causing an increase in the content of soil heavy metals such as Zn and Pb. The presence of Zn was an indicator element of petroleum combustion and steel smelting [50]. With the increase in coal consumption and the ban of leaded gasoline, this contribution gradually decreased, while that of coal combustion gradually increased. Presently, coal combustion has become the main source of atmospheric Pb [56]. Consequently, source two is attributed to the historical artisanal zinc smelting exhaust emissions and long-distance industrial coal combustion into the soil through atmospheric deposition. Further quantitative analysis with the UNMIX model revealed that the soil geochemical elements were mainly from the geological source (75.60) and atmospheric deposition (25.40) (Figure 5).

#### 3.4.3. Identification of Heavy Metal Sources by Pb-Cd Isotopic Composition

**Figure 7:**
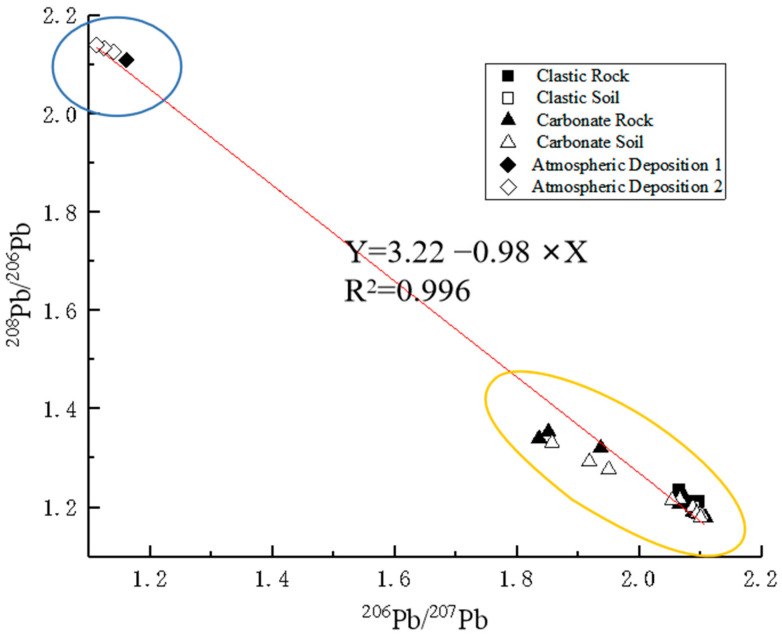
Scatter plot of lead isotope ratios of each material.

Figure 5 shows the differences in the isotope ratios in the various parent materials. The values for the ^208^Pb/^206^Pb and ^206^Pb/^207^Pb ratios were plotted for rock, atmospheric deposition, and soil in the study area to highlight the differences in Pb isotopic composition for different parent materials. For example, atmospheric deposition had the highest ^208^Pb / ^206^Pb ratio, which is higher than the ratio values reported in the study [35]. As shown in Figure 7, the order of the samples derived from the three parent materials from the lower right to the upper left were clastic rock and soil, carbonate rock and soil, and atmospheric deposition, which formed a fairly linear trend. The linear fitting was Y = 3.22 − 0.98 × X (R^2^ = 0.996). The isotopic composition of rock and soil was relatively concentrated in clastic areas, but fairly dispersed in carbonate areas, which is similar to the previous distribution of Pb. It shows that the carbonate rock weathering process is more complex than that of clastic rock. The carbonate rock weathering process has a large isotope fractionation effect. Wen Yubo’s [57] study of Pb isotope composition in karst areas found that carbonate rocks had a high ratio of ^206^Pb/^207^Pb, with an average composition of 1.49. Harlavan et al. [58] reported that the highest ratio of ^206^Pb/^207^Pb in marine carbonate rocks in Israel was 1.67. Table S2 shows that the average composition of ^206^Pb/^207^Pb in carbonate in this study was 1.53, which is consistent with the previous studies by Wen Yubo and Harlavan.

Based on the results of the UNMIX model and the Pb isotope scatter map, the weathering of geological parent rock and atmospheric deposition were the two main sources of heavy metals in soil. Metal isotopes have been utilized as effective means for tracing the source of heavy metals. For instance, Pb and Cd isotopes have been widely used to distinguish natural and anthropogenic sources of heavy metals along with improved analytical techniques and instrumental accuracy [59,60]. Generally, different potential sources have different compositions of Pb and Cd isotopes [61,62].

The sources were resolved using the isotopic binary model, and the results are shown in Table 9. Generally, the analysis showed that the isotope-resolved results are in agreement with those of the UNMIX. Factor one is the weathering process of geological parent rock. The mean values of the analytical results of the Pb isotope binary model were 78.92% and 58.27% for the carbonate and clastic areas, respectively. Similar to the study of Jia et al., the parent material was the most important source of Pb, and the contribution of the soil background to the topsoil Pb was the greatest, with a mean value of 74% [22]. It was found that geological sources are the main source of heavy metals in soil [63]. The secondary enrichment effect of weathering is the main means of heavy metal accumulation [3,10]. Factor two is the heavy metal input of atmospheric settlement into the soil. The mean value of the Pb isotope binary model analysis results is 21.08% in the carbonate rock area and 41.73 in the clastic area. At normal temperatures, Pb will vaporize into the atmosphere for long-distance transmission with the atmospheric deposition [22,64].

As shown in Table 10, the δ^114/110^Cd value in the carbonate sub-area soil samples ranged from −0.29‰ to +0.46‰, with a mean of 0.13‰. The δ^114/110^Cd value in the clastic sub-area soil samples ranged from −0.12‰ to +0.20‰, with a mean of 0.017‰. The atmospheric deposition samples had lower δ^114/110^Cd values (−0.314‰ ± 0.032‰; mean ±2r, which will be used subsequently) than any of the other samples. This suggests that Cd in atmospheric deposition is influenced by factors such as industrial smelting, gasoline combustion, and so on. The δ^114/110^Cd value in topsoil was remarkably higher than atmospheric deposition. This result was consistent with a previous study, which reported that the mean isotopic composition of polluted soils closer to the refinery plant show dramatically higher δ^114/110^Cd values compared to other samples [65]. The isotope composition of the soil samples in the study area is similar to the atmospheric settlement, such as the Ziliujing formation area (δ^114^/^110^Cd = −0.29). It shows that the long distance atmospheric deposition transmission is the important contribution of soil cadmium in the Ziliujing formation area. This is related to the factory operations and the evident spatial heterogeneity of Cd input sources in different research areas [6]. Therefore, the Cd isotope information can accurately identify and track each pollution source and contribution rate, and the determination of Cd isotope ratios in contaminated soil can provide valuable information, such as pollution control [6,21,66]. The analysis of lead isotopes revealed that the geological and long-distance transmission of sedimentation sources are the two main sources of heavy metals in the soil in the study area. According to the results shown in Table 10, in the carbonate area the geological source is 80.15%, and atmospheric deposition long-distance transmission is 19.85%, while in the clastic rock area, the geological source is 87.13% and atmospheric settlement long-distance transmission is 12.87%.

## 4. Conclusions

In this study, the rock- and soil-exposed carbonate area was assessed to explore the accumulation of geochemical elements and soil sources. The enrichment of heavy metals from parent rock weathered to soil was investigated in 10 formations in the carbonate and clastic areas of the Weining and Hezhang Counties in northwest Guizhou. The sources of soil geochemical elements were analyzed using a correlation analysis, a Pb-Cd isotopic composition, a Pb and isotope binary model, and the UNMIX model. The results revealed that the selected 13 geochemical elements (except for Si) in the soil of the study area are considerably higher than in the other soils in China. These findings reflect the unique character of the enrichment of heavy metals in karstic soil with high geochemical backgrounds under the same geological conditions. Elemental accumulations and losses in the clastic area are wide (0.20–11.38) compared with the carbonate area, suggesting a relative activity in the clastic area. A correlation analysis of the geochemical elements in soil shows that the sources of geochemical elements in the soil in the study area are complex. The isotopic composition of rock and soil are relatively concentrated in clastic areas but fairly dispersed in carbonate areas, which indicates that the carbonate rock weathering process is more complex and has a large isotope fractionation effect. Further quantitative analysis with the UNMIX model revealed that the soil geochemical elements are mainly from weathering of geological parent rocks and atmospheric deposition. Additionally, a comprehensive environmental risk assessment of the potential risk coefficient indicates that the overall geological high background level in soil in northwest Guizhou is at a slight risk level. The geoacumulation index showed that the heavy metals did not exceed the standard pollution reference in the clastic area. Furthermore, the single factor pollution index and geoaccumulation index revealed that the Cd at all sites exceeded the standard pollution reference, suggesting that additional accumulation of heavy metals occurred during the rock weathering process. This study highlights that a comprehensive and reliable assessment of the status of soil heavy metal contamination in rock weathering development soils is imperative for environmental monitoring, remediation, and decision making.

## Figures and Tables

**Table 1:** Samples of formations and rock information in the study area.

Stratum	Formation	Rock Types
Triassic	Erqiao Formation	Clastic rock
	Feixianguan Formation	
Permian	Xuanwei Formation	
	Longtan Formation	
	Maokou Formation	Carbonate rocks
	Maping Formation	
Carbonic	Huanglong Formation	
Triassic	Guanling Formation	
	Jialingjiang Formation	
Jurassic	Ziliujing Formation	
	Suining Formation	

**Table 2:** Criteria of pollution grade of soil heavy metals.

Single-Factor Pollution Index		Geoaccumulation Index			Potential Ecological Risk Index		
*Pi*	Pollution Classification	*Igeo*	Grade	Pollution Classification	*Ei*	*RI*	Ecological Grade
*P_i_* ≤ 1	Unpolluted	*Igeo* ≤ 0	0	Unpolluted	*E_i_* ≤ 40	*RI* ≤ 150	Low risk
1 < *P_i_* ≤ 2	Warning	0 < *Igeo* ≤ 1	1	Slightly polluted	40 < *E_i_* ≤ 80	150 < *RI* ≤ 300	Moderate risk
2 < *P_i_* ≤ 3	Slightly polluted	1 < *Igeo* ≤ 2	2	Moderately polluted	80 < *E_i_* ≤ 160	300 < *RI* ≤ 600	High risk
3 < *P_i_* ≤ 5	Moderately polluted	2 < *Igeo* ≤ 3	3	Moderately-heavily polluted	160 < *E_i_* ≤ 320	600 < *RI* < 1200	Very high risk
*P_i_* > 5	Heavily polluted	3 < *Igeo* ≤ 4	4	Heavily polluted	*E_i_* < 320	*RI* > 1200	Considerable risk
		4 < *Igeo* ≤ 5	5	Heavily-extremely polluted			
		*Igeo* > 5	6	Extremely polluted			

**Table 3:** Statistical summary of the geochemical parameters in rocks.

Element	Units	Rocks in Carbonate Sub-Area (*n* = 39)						Rocks in Clastic Sub-Area (*n* = 30)						
		Min	Median	Max	Mean	SD	CV (%)	Min	Median	Max	Mean	SD	CV (%)	Abundances
Cd	mg/kg	0.07	0.20	4.74	0.49	0.90	184.24	0.08	0.165	1.52	0.37	0.43	117.84	0.12
As	-	0.57	3.48	374.4	17.40	62.86	361.27	0.31	1.56	4.04	1.51	0.86	57.17	3.53
Pb	-	0.27	8.24	1144	42.68	184.42	432.06	0.69	8.79	24.9	9.70	6.84	70.58	9.02
Cr	-	25.10	65.25	823.60	97.71	127.20	130.18	84.40	227.8	698.3	297.30	194.26	65.34	9.49
Cu	-	1.37	15.60	1790.80	115.62	325.69	281.69	48.40	169.6	614.2	214.64	144.76	67.45	5.69
Ni	-	4.88	23.00	2298.30	91.53	368.76	402.88	21.90	90.65	130.00	84.27	31.78	37.72	6.09
Zn	-	1.58	31.35	1050.70	68.76	164.33	238.99	24.10	118.60	160.30	112.28	36.62	32.61	21.19
SiO_2_	wt%	2.01	41.04	92.79	42.68	23.60	55.31	9.30	35.89	79.95	42.25	22.56	53.40	10.07
Al_2_O_3_	-	1.40	13.66	41.06	14.86	10.17	68.40	0.32	17.40	30.85	14.57	9.12	62.61	1.77
TFe_2_O_3_	-	1.12	3.65	41.80	5.94	7.53	126.75	5.44	11.80	24.85	12.34	5.74	46.52	0.50
MgO	-	0.3	1.82	8.28	2.61	2.23	85.47	0.50	3.13	4.25	2.41	1.49	61.75	6.36
CaO	-	0.21	7.67	67.58	24.27	27.12	111.77	0.13	0.23	5.12	0.74	1.34	181.81	39.7
K_2_O	-	0.20	1.00	5.13	1.58	1.43	90.19	0.49	1.03	2.96	1.24	0.84	68.28	0.54

**Table 4:** Statistical table of heavy metal concentration of soil in different formations.

Region	Formation	Cd	As	Pb	Cr	Cu	Ni	Zn
	Background value	0.4668	20.1	33.4	139.6	66.58	56.89	111.4
Carbonate sub-area (*n* = 44)	Suining Formation	1.74 (±0.70)	17.98 (±1.61)	54.20 (±25.25)	78.40 (±12.54)	41.03 (±11.97)	39.43 (±5.84)	188.83 (±86.50)
	Ziliujing Formation	0.49 (±0.40)	9.34 (±1.03)	29.08 (±3.98)	83.11 (±21.49)	34.30 (±3.76)	35.59 (±6.40)	87.05 (±16.57)
	Guanling Formation	0.65 (±0.39)	24.05 (±7.38)	29.23 (±12.76)	222.61 (±112.60)	85.38 (±35.03)	81.49 (±30.24)	113.03 (±21.64)
	Jialingjiang Formation	0.81 (±0.23)	9.24 (±2.00)	63.42 (±19.07)	236.25 (±34.74)	136.35 (±15.91)	99.28 (±10.64)	223.52 (±24.11)
	Maokou Formation	1.15 (±0.47)	25.27 (±13.34)	35.59 (±6.86)	134.74 (±17.74)	115.18 (±81.27)	79.21 (±49.30)	139.86 (±20.90)
	Maping Formation	1.95 (±1.82)	27.35 (±2.60)	65.43 (±14.04)	131.67 (±4.62)	35.88 (±2.89)	51.40 (±6.41)	148.03 (±39.99)
	Huanglong Formation	5.21 (±0.71)	46.30 (±0.62)	68.12 (±11.18)	216.00 (±4.56)	43.77 (±6.61)	96.93 (±5.71)	244.33 (±31.30)
Clastic Sub-area (*n* = 16)	Erqiao Formation	1.59 (±0.25)	66.75 (±16.44)	28.58 (±4.94)	147.48 (±61.10)	205.65 (±48.49)	257.80 (±38.40)	391.98 (±50.33)
	Feixianguan Formation	2.42 (±0.75)	67.59 (±9.37)	210.20 (±19.50)	351.35 (±18.73)	199.65 (±17.94)	491.84 (±21.91)	567.21 (±39.93)
	Longtan Formation	7.66 (±3.84)	8.47 (±2.42)	51.10 (±19.69)	292.93 (±42.21)	115.85 (±15.50)	98.83 (±7.94)	439.43 (±65.65)
	Xuanwei Formation	2.54 (±0.92)	7.28 (±1.19)	34.48 (±9.01)	199.38 (±61.17)	278.75 (±56.67)	123.58 (±18.54)	222.25 (±55.79)

Note: The data are the mean ± standard deviation.

**Table 5:** Statistical summary of the geochemical parameters in soil.

	Units	Surface Soil in Carbonate Sub-Area (*n* = 39)						Surface Soil in Clastic Sub-Area (*n* = 30)						Background Value
		Min	Median	Max	Mean	SD	CV (%)	Min	Median	Max	Mean	SD	CV (%)	
Cd	mg/kg	0.31	1.11	8.37	1.90	2.10	125.74	0.11	1.17	6.65	1.61	1.52	77.54	0.4668
As	-	6.30	12.85	45.90	19.93	14.58	73.41	5.64	8.79	13.90	9.25	2.24	187.84	20.10
Pb	-	16.00	32.10	95.20	43.62	23.44	51.62	18.00	31.80	216.10	48.91	52.84	98.49	33.40
Cr	-	60.40	133.80	379.00	155.80	86.12	58.44	74.10	139.50	306.20	166.20	81.86	60.21	139.60
Cu	-	23.00	46.05	270.90	72.62	56.15	78.11	35.50	120.40	419.90	154.81	116.65	69.39	66.58
Ni	-	28.90	55.35	196.50	69.88	36.88	54.03	28.70	77.85	152.90	84.18	40.72	262.53	56.89
Zn	-	75.40	135.30	323.30	155.96	67.50	44.45	75.60	122.55	378.40	161.31	86.46	124.20	111.40
pH	-	4.69	6.53	8.03	6.43	0.91	14.00	5.45	6.25	7.74	6.43	0.70	10.90	
SiO_2_	wt%	4.91	39.05	121.35	44.46	21.91	44.52	0.43	41.94	130.52	46.95	23.29	31.44	58.00
Al_2_O_3_	-	3.12	22.47	39.23	21.86	6.92	32.15	0.08	1.30	10.20	1.79	1.86	101.73	14.11
TFe_2_O_3_	-	5.70	9.74	16.58	10.27	3.40	35.33	5.86	13.66	111.67	17.22	19.33	109.75	9.82
MgO	-	0.22	2.11	6.02	2.32	1.43	74.54	0.30	18.23	31.15	18.91	7.59	37.18	1.47
CaO	-	0.18	1.06	2.55	1.03	0.60	60.94	0.23	0.63	3.37	0.75	0.59	56.16	0.93
K_2_O	-	0.47	2.18	5.46	2.45	1.30	58.30	0.06	0.99	5.54	1.52	1.40	87.76	1.27

**Table 6:** Enrichment coefficient and Q value of soil and rock.

	Index	Cd	As	Pb	Cr	Cu	Ni	Zn	SiO_2_	Fe_2_O_3_	MgO	Al_2_O_3_	CaO	K_2_O
Carbonate rock	Mean	0.49	17.40	42.68	97.71	115.62	91.53	68.76	42.68	14.86	5.94	2.61	24.27	1.58
	Abundances	0.12	3.53	9.02	9.49	5.69	6.09	21.19	10.07	0.5	6.36	1.77	39.7	0.54
	enrichment coefficient	4.08	4.93	4.73	10.30	20.32	15.03	3.24	4.24	29.72	0.93	1.47	0.61	2.93
	Enrichment	Strong Enrichment	Strong Enrichment	Strong Enrichment	Strong Enrichment	Strong Enrichment	Strong Enrichment	Strong Enrichment	Strong Enrichment	Strong Enrichment	Loss	Strong Enrichment	Strong Loss	Strong Enrichment
Carbonate soil	Mean	1.51	18.39	41.59	147.91	67.22	64.55	143.88	45.45	21.63	9.82	2.35	0.94	2.73
	Abundances	0.467	20.10	33.40	139.6	66.58	56.89	111.4	58.00	14.11	9.82	1.47	0.93	1.27
	enrichment coefficient	3.23	0.91	1.25	1.06	1.01	1.13	1.29	0.78	1.53	1.00	1.60	1.01	2.15
	Enrichment	Strong Enrichment	Loss	Strong Enrichment	Moderate Enrichment	Moderate	Enrichment	Strong Enrichment	Strong Loss	Strong Enrichment	Moderate	Strong Enrichment	Moderate	Strong Enrichment
	Q value	3.08	1.06	0.97	1.51	0.58	0.71	2.09	1.06	1.46	1.65	0.90	0.04	1.73
Rock	Mean	0.37	1.51	9.70	297.30	214.64	84.27	112.28	42.25	14.57	12.34	2.41	0.74	1.24
	Abundances	0.09	5.10	17.54	39.16	15.91	17.59	50.48	73.8	2.39	1.21	10.59	2.22	2.34
	enrichment coefficient	4.11	0.30	0.55	7.58	13.45	4.79	2.22	0.57	6.11	10.17	0.23	0.33	0.53
	Enrichment	Strong Enrichment	Strong Loss	Strong Loss	Strong Enrichment	Strong Enrichment	Strong Enrichment	Strong Enrichment	Strong Loss	Strong Enrichment	Strong Enrichment	Strong Loss	Strong Loss	Strong Loss
Soil	Mean	1.66	17.18	79.64	257.07	170.97	333.49	287.63	42.90	19.50	22.59	2.43	0.86	1.27
	Abundances	0.47	20.10	33.40	139.60	66.58	56.89	111.40	58.00	14.11	9.82	1.47	0.93	1.27
	enrichment coefficient	3.53	0.85	2.83	1.84	2.57	5.86	2.58	0.74	1.38	2.30	1.65	0.92	1.00
	Enrichment	Strong Enrichment	Strong Loss	Strong Enrichment	Strong Enrichment	Strong Enrichment	Strong Enrichment	Strong Enrichment	Strong Loss	Strong Enrichment	Strong Enrichment	Strong Enrichment	Strong Loss	Moderate
	Q value	4.49	11.38	8.21	0.86	0.80	3.96	2.56	1.02	1.34	1.83	1.01	1.16	1.02

**Table 7:** Evaluated results from the single factor index and the geoaccumulation pollution index for soil heavy metals.

Region	Elements	Pollution Index	Sampling (%)				
			Unpolluted	Warning	Slightly Polluted	Moderately Polluted	Heavily Polluted
Carbonate sub-area (*n* = 39)	Cd	*P_i_*	18.75	15.63	31.25	9.38	25.00
	As	*P_i_*	68.75	12.50	18.75	-	-
	Pb	*P_i_*	53.13	25.00	21.88	-	-
	Cr	*Pi*	65.63	21.88	12.50	-	-
	Cu	*P_i_*	68.75	18.75	9.38	3.13	-
	Ni	*P_i_*	53.13	40.63	3.13	3.13	-
	Zn	*P_i_*	28.13	59.38	12.50	-	-
Clastic sub-area (*n* = 30)	Cd	*P_i_*	17.86	25.00	28.57	3.57	25.00
	As	*P_i_*	100.00	-	-	-	-
	Pb	*P_i_*	67.86	21.43	-	-	10.71
	Cr	*P_i_*	50.00	35.71	14.29	-	-
	Cu	*P_i_*	32.14	25.00	14.29	25.00	3.57
	Ni	*P_i_*	46.43	21.43	32.14	-	-
	Zn	*P_i_*	50.00	14.29	32.14	3.57	-
Carbonate sub-area (*n* = 39)	Cd	*Igeo*	90.63	9.38	-	-	
	As	*Igeo*	68.75	31.25	-	-	
	Pb	*Igeo*	78.13	21.88	-	-	
	Cr	*Igeo*	78.13	21.88	-	-	
	Cu	*Igeo*	71.88	28.13	-	-	
	Ni	*Igeo*	65.63	31.25	3.13	-	
	Zn	*Igeo*	62.50	37.50	-	-	
Clastic sub-area (*n* = 30)	Cd	*Igeo*	100.00	-	-	-	
	As	*Igeo*	100.00	-	-	-	
	Pb	*Igeo*	96.43	3.57	-	-	
	Cr	*Igeo*	78.57	21.43	-	-	
	Cu	*Igeo*	39.29	32.14	25.00	3.57	
	Ni	*Igeo*	50.00	50.00	-	-	
	Zn	*Igeo*	64.29	32.14	3.57	-	

**Table 8:** Correlation analysis of geochemical elements of soil.

	pH	Cd	As	Pb	Cr	Cu	Ni	Zn	SiO_2_	Al_2_O_3_	Fe_2_O_3_	MgO	CaO
pH	1												
Cd	−0.20	1											
As	−0.39 **	0.60 **	1										
Pb	0.07	0.30 *	0.16	1									
Cr	−0.08	0.26	−0.10	0.42 **	1								
Cu	−0.28	0.01	−0.36 *	−0.01	0.48 **	1							
Ni	−0.16	0.34 *	−0.11	0.33 *	0.86 **	0.78 **	1						
Zn	−0.10	0.75 **	0.20	0.61 **	0.58 **	0.33 *	0.65 **	1					
SiO_2_	0.28	−0.29	−0.20	−0.16	−0.20	−0.20	−0.29	−0.22	1				
Al_2_O_3_	−0.25	−0.02	0.23	−0.05	−0.08	0.03	−0.05	−0.07	−0.07	1			
Fe_2_O_3_	−0.34 *	0.08	−0.26	0.07	0.57 **	0.92 **	0.84 **	0.37 *	−0.34 *	0.05	1		
MgO	0.11	0.04	−0.15	0.42 **	0.41 **	0.02	0.23	0.34 *	−0.28	−0.12	0.07	1	
CaO	0.46 **	0.17	−0.30 *	0.10	0.44 **	0.09	0.34 *	0.50 **	0.04	−0.28	0.10	0.44 **	1

** At level 0.01 (double-tail), the correlation was significant. * At level 0.05 (double-tail), the correlation was significant.

**Table 9:** Cd content and Cd isotopic compositions of rock and soils.

Region	Famation	Cd (mg/kg)	δ^114^/^110^Cd
Rock in study area	Suining Formation	0.40 ± 0.23	−0.07 ± 0.06
	Guanling Formation	0.14 ± 0.38	0.60 ± 0.08
	Feixianguan Formation	0.54 ± 0.14	0.15 ± 0.05
	Jialingjiang Formation	0.24 ± 0.18	0.17 ± 0.08
	Maokou Formation	0.18 ± 0.04	0.18 ± 0.10
	Huanglong Formation	0.39 ± 0.18	0.61 ± 0.08
Soil in carbonate sub-area	Suining Formation	1.74 ± 0.70	0.16 ± 0.09
	Ziliujing Formation	0.49 ± 0.40	−0.29 ± 0.08
	Guanling Formation	0.65 ± 0.39	0.25 ± 0.04
	Jialingjiang Formation	0.81 ± 0.23	0.14 ± 0.06
	Maokou Formation	1.15 ± 0.47	0.46 ± 0.05
	Huanglong Formation	5.21 ± 0.71	0.06 ± 0.07
Soil in clastic sub-area	Longtan Formation	7.66 ± 3.84	−0.12 ± 0.06
	Xuanwei Formation	2.54 ± 0.92	0.20 ± 0.09
	Erqiao Formation	1.59 ± 2.25	−0.03 ± 0.05
	Atmospheric deposition	0.69 ± 0.21	−0.314 ± 0.03

**Table 10:** Analysis results of the sources of heavy metals in soil.

Region	Analytic Method	Geological Source	Atmospheric Deposition
All samples	Unmix model	75.60	24.40
Carbonate sub-area	^206^Pb/^207^Pb	78.02	21.98
Carbonate sub-area	^208^Pb/^206^Pb	79.81	20.19
Clastic sub-area	^206^Pb/^207^Pb	55.00	45.00
Clastic sub-area	^208^Pb/^206^Pb	61.54	38.46
Carbonate sub-area	^114^Cd/^110^Cd	80.15	19.85
Clastic sub-area	^114^Cd/^110^Cd	87.13	12.87

## Data Availability

The datasets utilized in this study are available upon request from the corresponding author.

## Supplementary Materials

The following supporting information can be downloaded at: https://www.mdpi.com/article/10.3390/ijerph191912478/s1, Table S1. Statistical table of heavy metal concentration of rock in different Formation. Table S2. Pb isotopic compositions of rock and soils.
