# Supplementary material for: Contamination Evaluation and Source Analysis of Heavy Metals in Karst Soil Using UNMIX Model and Pb-Cd Isotopes
Source: Int J Environ Res Public Health. 2022 Sep 30;19(19):12478. doi: 10.3390/ijerph191912478 (PMC9566772; doi:10.3390/ijerph191912478)
Supplement: Supplementary file 1 [file ijerph-19-12478-s001.zip › ijerph-1895489-supplementary.pdf]

Table S1. Statistical table of heavy metal concentration of rock in different Formation.

Region	Formation	Cd	As	Pb	Cr	Cu	Ni	Zn
	Abundance	0.12	3.53	9.02	9.49	5.69	6.09	21.19
Carbonate sub–area (*n* = 39)	Suining Formation	0.40 (±0.23)	4.24 (±2.63)	10.70 (±5.46)	64.88 (±21.24)	23.46 (±22.10)	22.04 (±11.16)	41.50 (±21.59)
	Ziliujing Formation	0.15 (±0.14)	11.01 (±13.92)	14.59 (±14.76)	66.44 (±25.70)	30.32 (±28.62)	28.35 (±12.08)	52.72 (±33.41)
	Guanling Formation	0.14 (±0.38)	1.25 (±2.17)	2.64 (±7.42)	16.13 (±18.85)	11.74 (±64.27)	5.77 (±56.27)	22.38 (±71.92)
	Jialingjiang Formation	0.24 (±0.18)	3.67 (±1.13)	6.64 (±6.63)	126.88 (±97.03)	65.34 (±39.57)	51.35 (±19.35)	55.46 (±13.82)
	Maokou Formation	0.18 (±0.04)	2.19 (±1.03)	5.70 (±3.98)	57.50 (±21.49)	6.71 (±3.76)	14.70 (±6.40)	27.20 (±16.57)
	Maping Formation	0.12 (±0.03)	3.48 (±2.00)	15.50 (±19.07)	37.60 (±84.74)	1.37 (±15.91)	8.99 (±10.64)	12.00 (±4.11)
	Huang Long Formation	0.39 (±0.18)	2.91 (±0.97)	7.45 (±5.95)	59.78 (±15.67)	5.39 (±3.98)	12.06 (±4.49)	8.60 (±5.10)
Clastic sub–area (*n* = 30)	Erqiao Formation	0.38(±0.24)	2.17(±2.53)	7.42(±4.16)	128.85(±55.89)	64.27(±14.07)	56.27(±17.42)	71.92(±26.49)
	Feixianguan Formation	0.54(±0.14)	1.88(±1.96)	6.01(±13.50)	85.77(±39.40)	53.59(±26.10)	43.04(±84.90)	54.13(±31.30)
	Longtan Formation	0.18(±0.14)	3.28(±1.92)	9.76(±1.76)	26.50(±5.70)	11.90(±2.62)	19.70(±2.08)	23.70(±3.41)
	Xuanwei Formation	0.19(±0.14)	0.73(±0.53)	1.98(±1.16)	260.8(±55.89)	389.10(±14.07)	89.50(±17.42)	123.10(±26.49)

Note: The data are the mean ± standard deviation.

Table S2. Pb isotopic compositions of rock and soils.

Region	Formation	$^{208}Pb/^{204}Pb$	$^{207}Pb/^{204}Pb$	$^{206}Pb/^{204}P$	$^{208}Pb/^{206}Pb$	$^{206}Pb/^{207}Pb$
Rock in clastic sub–area	Erqiao Formation	38.8933	16.8149	41.1400	0.9454	2.4466
	Xuanwei Formation	39.9002	15.6426	19.3213	2.0651	1.2352
	Feixianguan Formation	39.5014	15.6136	18.9312	2.0866	1.2125
	Longtan Formation	39.6935	15.6149	18.9212	2.0978	1.2117
Rock in carbonate sub–area	Maping Formation	39.2996	15.7609	19.0203	2.0662	1.2068
	Suining Formation	38.9463	15.6632	18.6477	2.0885	1.1905
	Maokou Formation	40.6272	15.8844	20.9692	1.9375	1.3201
	Ziliujing Formation	39.3040	15.7105	18.9526	2.0738	1.2064
	Huanglong Formation	38.9663	15.8449	21.2197	1.8363	1.3392
	Guanling Formation	39.4806	15.7609	21.3180	1.852	1.3526
	Jialingjiang Formation	39.5240	15.6321	19.0447	2.0753	1.2183
Soil in clastic sub–area	Erqiao Formation	39.2437	15.7920	20.9097	1.8768	1.3241
	Xuanwei Formation	39.2806	15.6485	18.7947	2.09	1.2011
	Feixianguan Formation	39.0662	15.6785	18.6443	2.0953	1.1892
	Longtan Formation	39.0636	15.7075	18.6558	2.0939	1.1877
Soil in carbonate sub–area	Maping Formation	39.2640	15.7469	19.1151	2.0541	1.2139
	Suining Formation	38.7738	15.6506	18.4581	2.1006	1.1794
	Maokou Formation	39.1239	15.7784	20.3896	1.9188	1.2923
	Ziliujing Formation	39.4809	15.7175	19.1003	2.067	1.2152
	Huanglong Formation	39.1435	15.8424	21.0719	1.8576	1.3301
	Guanling Formation	39.2445	15.6661	18.7949	2.088	1.1997
	Jialingjiang Formation	39.1469	15.7323	18.6099	2.1035	1.1829
